# FrAnTK: a Frequency-based Analysis ToolKit for efficient exploration of allele sharing patterns in present-day and ancient genomic datasets

**DOI:** 10.1093/g3journal/jkab357

**Published:** 2021-10-13

**Authors:** J Víctor Moreno-Mayar

**Affiliations:** Lundbeck Foundation GeoGenetics Centre, GLOBE Institute, University of Copenhagen, 1350 Copenhagen, Denmark

**Keywords:** population genomics, allele sharing statistics, user-friendly pipeline, ancient DNA, python, R, perl

## Abstract

Present-day and ancient population genomic studies from different study organisms have rapidly become accessible to diverse research groups worldwide. Unfortunately, as datasets and analyses become more complex, researchers with less computational experience often miss their chance to analyze their own data. We introduce FrAnTK, a user-friendly toolkit for computation and visualization of allele frequency-based statistics in ancient and present-day genome variation datasets. We provide fast, memory-efficient tools that allow the user to go from sequencing data to complex exploratory analyses and visual representations with minimal data manipulation. Its simple usage and low computational requirements make FrAnTK ideal for users that are less familiar with computer programming carrying out large-scale population studies.

## Introduction 

Recent advances in DNA retrieval and sequencing techniques have made it possible to obtain whole-genome (Bergström *et al.* 2020; Margaryan *et al.* 2020) and genome-wide (Olalde *et al.* 2018) data from hundreds of present-day and ancient individuals in single studies. Together with the development of statistics for hypothesis testing, *e.g.*, *f*-statistics (Patterson *et al.* 2012), these large datasets have allowed us to characterize the genetic structure and demographic history of diverse populations with unprecedented resolution. Genomic population history studies often rely on comparing data from the population(s) of interest with reference variation datasets from other ancient and contemporary populations. This comparison is usually performed by applying methods such as principal components analysis (Patterson *et al.* 2006), multidimensional scaling (Malaspinas *et al.* 2014), model-based clustering (Alexander *et al.* 2009), and hypothesis testing through *f*-statistics (Patterson *et al.* 2012). The latter have been particularly useful as they can be used both for exploratory analyses, *e.g.*, measuring shared drift between pairs of populations, and for formal hypothesis testing, *e.g.*, testing for admixture and estimating admixture proportions. Importantly, *f*-statistics can be computed from pseudo-haploid calls from ancient DNA (aDNA) data where calling diploid genotypes is challenging due to low-depth and increased error (but see Günther and Nettelblad 2019). This strategy is not confined to human evolution research, but it has been successfully applied to other study organisms, *e.g.*, maize (Ramos-Madrigal *et al.* 2016), horses (Gaunitz *et al.* 2018), and canids (Ramos-Madrigal *et al.* 2021).

Over the last few years, ancient and present-day population genomic sequencing has become accessible to more research groups worldwide. However, many data analysis tools remain accessible only to users with more experience in computer programming, thus creating a disconnect between researchers in charge of data generation, researchers with data analysis expertise, and researchers from diverse disciplines involved in results interpretation. Whereas this might be unavoidable in some cases, simplifying routine exploratory data analyses into user-friendly tools could facilitate the contribution of researchers with less computational experience to genomic data analysis and interpretation. We introduce FrAnTK, a fast, user-friendly toolkit that allows users to easily combine their sequencing data with reference datasets and compute and visualize allele frequency-based statistics routinely used in population genomic studies. FrAnTK is mainly aimed at users with limited programming experience, but we anticipate it will also be helpful to experienced users who seek to streamline exploratory analyses and visualization.

## Methods

**Figure 1:**
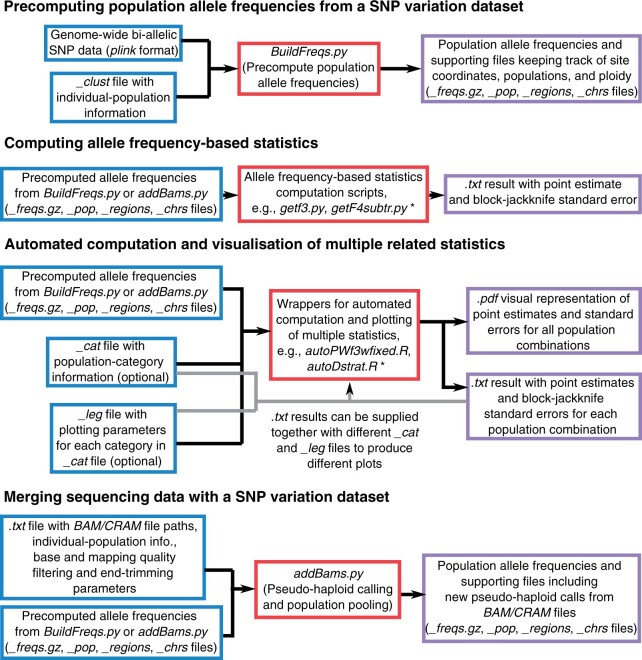
Overview of the main FrAnTK functionalities, their input and output files. Input files are shown in blue, operations performed by FrAnTK are shown in red and output files are shown in purple. *See Supplementary Table S1 and github.com/morenomayar/FrAnTK#SecComputingA SingleStatistic for a full list of the analyses implemented in FrAnTK.

FrAnTK contains tools with four main functionalities summarized in Figure 1: (1) precomputing the population allele frequencies from a SNP variation dataset, (2) computing allele frequency-based statistics, (3) visualizing multiple related statistics, and (4) merging sequencing data with a reference SNP variation dataset. We provide a description of the main tools included in FrAnTK in Supplementary Table S1.

We start with a plink file (Chang *et al.* 2015) containing genome-wide bi-allelic SNP data from multiple individuals from different populations. In this case, we rely on plink files as they have been widely used by researchers in the field for over a decade (see the practical example included below for a note on how to go from vcf to plink). To speed up the subsequent computation of test statistics and to substantially reduce computational resource requirements (see benchmark below), we first compute the allele frequencies for each SNP site for each population. We store these frequencies in a .gz compressed file, accompanied by three more files that keep track of SNP site coordinates and population names and ploidy (see file format description in Supplementary Information).

We supply a set of scripts for computing allele frequency-based statistics (Supplementary Table S1). These scripts take the precomputed allele frequencies as input and return a point estimate together with its standard error, estimated through a weighted block-jackknife procedure (Patterson *et al.* 2012). In this release, we provide scripts for computing average pairwise distances, *f3*, *f4*, and *D*-statistics, *f4*-ratios (Patterson *et al.* 2012), admixture/contamination-corrected *f4*-statistics (Reich *et al.* 2012), minor allele count-stratified *D*-statistics (Prüfer *et al.* 2014), and enhanced *D*-statistics that rely on specific ascertainment schemes to detect faint allele sharing patterns (Meyer *et al.* 2012). All analyses can be restricted to transversion polymorphisms to reduce the effect of aDNA *postmortem* damage (Briggs *et al.* 2007). These scripts can be run in parallel as they keep memory usage to a minimum by processing one SNP at a time. Moreover, they can be easily modified to accommodate additional analyses. We note that an implementation of some of these analyses, *e.g.*, admixture/contamination-corrected *f4*-statistics (Reich *et al.* 2012), enhanced *D*-statistics (Meyer *et al.* 2012), has not been made available elsewhere or depend on additional data manipulation, which hinders their widespread usage.

To supplement the scripts for computing single statistics, we provide multi-threaded wrappers for automated computation of multiple related statistics (Supplementary Table S1). A common exploratory analysis is to assess the genetic relationship between a fixed test population and a set of reference populations. For instance, we can compute all possible *f3*-statistics of the form *f3(TestPop, X; Outgroup)*, to explore what is the population *X* in the reference dataset that shares the most drift with the test population. Our wrapper scripts make it possible to run this kind of analysis with a single command and support average pairwise distances, *f_3_*, *f_4_*, “basic” *D* and enhanced *D*-statistics. In addition, we include wrapper scripts for computing admixture/contamination-corrected *f_4_*-statistics over a range of admixture/contamination proportions and minor allele count-stratified *D*-statistics over a specified range of minor allele counts.

**Figure 2:**
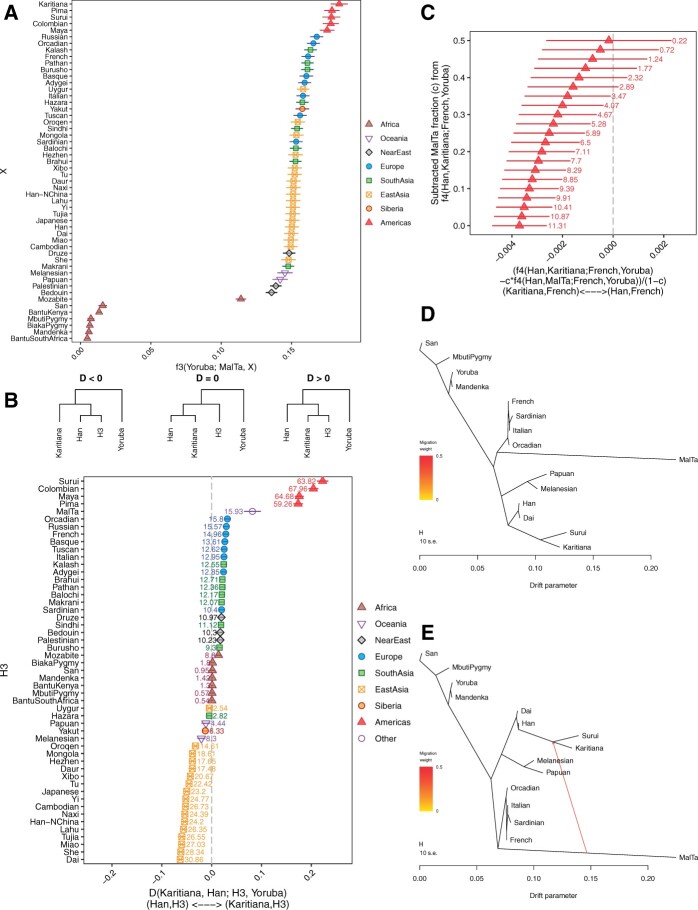
Visual representation of the worldwide allele sharing patterns of the ∼24ka Mal'ta individual produced by FrAnTK. (A) Shared genetic drift between the Mal'ta individual and present-day populations in the HGDP dataset. We show the *autof3wfixed.R* wrapper results for *f_3_(Mal'ta, X; Yoruba)*. Error bars represent 1.96 standard errors. (B) D-statistics exploring non-East Asian admixture in the Karitiana (an Indigenous American population). We show the *autoDwfixed.R* wrapper results for *D(Karitiana, Han; X, Yoruba)*. Error bars represent 3.3 standard errors. |*Z*|-scores are shown next to each point. (C) Mal'ta-related admixture-corrected *f_4_*-statistics of the form *f_4_(Han, Karitiana; French, Yoruba)*. For each statistic, we subtracted *f_4_(Han, Mal'ta; French, Yoruba)* weighted by an admixture proportion *c* ranging between 0% and 50% (*y*-axis) using the *autof4subtr.R* wrapper. Error bars represent 3.3 standard errors. (D,E) *TreeMix* admixture graphs (as output by the *TreeMix* software) relating the ancient Mal'ta individual and a set of worldwide present-day populations. We fit admixture graphs with zero and one admixture edges.

The wrapper scripts also provide automated plotting functionality, which allows users to create visual representations of classical exploratory analyses (*e.g.*, Figure 2, A–C). For every run, we automatically produce a plot with default plotting parameters. The user can easily customize this plot by providing simple files that map populations to categories with specific plotting parameters (see usage example below).

FrAnTK includes a single-command tool for merging sequencing data in BAM/CRAM format (Li *et al.* 2009) with the precomputed allele frequency files. For a given BAM/CRAM file, we sample one random allele at every SNP position included in the reference dataset to generate pseudo-haploid calls (calls giving rise to tri-allelic sites are set to missing). Reads and bases can be filtered according to their mapping and base quality. Additionally, the user can request a given number of bases to be ignored from both ends of each read to reduce aDNA *postmortem* damage-related error (Briggs *et al.* 2007). This merging approach is intended for cases where the user has access to low-to-intermediate-depth genome-wide sequencing data from a small sample of individuals, where reliable diploid genotype calling is not feasible, *e.g.*, aDNA data (Nielsen *et al.* 2011; Günther and Nettelblad 2019). Thus, we restrict the analyses to known segregating sites present in the reference variation dataset (precomputed allele frequencies). For cases where sequencing data are available for a large enough sample, such that joint SNP calling is feasible using tools such as ANGSD (Korneliussen *et al.* 2014), the user can use the tools in FrAnTK to create pseudo-haploid calls on the previously identified segregating sites, without recurring to genotype reference data.

FrAnTK is designed to be accessible, from installation to advanced usage, to researchers with less computational experience and to be applicable to a diverse range of projects. The tools in FrAnTK are implemented in python (python2 and python3 are supported), R and perl and depend only on standard tools and libraries that are commonly present in Unix-like setups in the field of population genomics: plink (Chang *et al.* 2015), samtools (Li *et al.* 2009) and the doParallel and ggplot2 (Wickham 2016) R libraries. Moreover, FrAnTK can be used on any genome-wide dataset from any study organism with a genome assembly contiguousness that allows for proper block-jackknife sampling.

## Results

To assess the performance of our toolkit, we computed a number of statistics on a whole-genome reference dataset using FrAnTK and admixtools (Patterson *et al.* 2012), and recorded the running time and memory usage in each case. Although multiple tools for computing *f*- and other site-statistics are available, *e.g.*, popstats (Skoglund *et al.* 2015), we use admixtools for benchmarking as it is the most commonly used peer-reviewed tool in the field. Moreover, admixtools is also called internally by the popular admixr wrapper (Petr *et al.* 2019). We used the SGDP Team C dataset (Mallick *et al.* 2016), which contains diploid genotypes for 33,512,001 bi-allelic SNPS from 345 individuals from 164 populations. We computed the 161 possible *D*-statistics of the form *D(French, Sardinian; X, Ju-hoan_North)*, which we distributed over 20 threads (Intel Xeon Gold 2.10 GHz CPU). Using the qpDstat (v712) program from admixtools, the task was completed in 5.1 h, with a peak memory usage of ∼520 GB (∼26 GB per thread). Using the automated *D*-statistic wrapper in FrAnTK (*autoDwfixed.R*), the task was completed in 2.2 h, with a peak memory usage of ∼5.8 GB (∼287 MB per thread). We attribute these gains to two key features: (1) precomputing the allele frequencies to speed up subsequent computation and (2) processing one site at a time instead of loading the whole dataset onto memory. In this example, the admixtools-based approach would require the user to prepare a set of input files and distribute the parallel processes across different threads. Once all processes have run, the user would have to parse separate results and use a custom script for visualizing the results. By contrast, by using FrAnTK, the user can go from the initial data to a visual representation of the results by running two one-line commands.

### Practical example

We present a practical example on how to use some of the different tools included in FrAnTK to explore the genetic ancestry of an ancient human whose genome has been sequenced to an average depth of coverage of 0.1X. We run the example on a compute node with 40 cores (Intel Xeon Gold 2.10 GHz CPU). We use the HGDP SNP array dataset (Li *et al.* 2008) as a reference and a subset of the 1X genome from an individual that lived ∼24 ka in the Central South Siberian site of Mal'ta (Raghavan *et al.* 2014). The data for running this example can be downloaded using *wget*.

wget https://sid.erda.dk/share_redirect/E48FQXKjCe \

-O FrAnTKPrcaticalExampleData.tar.gz

The HGDP SNP array dataset contains diploid genotypes for 644,088 bi-allelic SNPS from 938 individuals from 53 worldwide populations. We start by precomputing the population allele frequencies of the HGDP reference dataset. This procedure was completed in 2.3 min.

#get the number of populations in the _clust file *n* = `cut -f 3 HGDP_hg19_genotypes_clust | sort | uniq | wc -l `



#precompute population allele frequencies

frantk BuildFreqs plinkpref=HGDP_hg19_genotypes \

clustfile=HGDP_hg19_genotypes_clust npops="$n" \

prefout=HGDP_hg19_genotypes_f

In case the reference data were provided in a vcf file instead of a plink file, we can obtain a suitable plink file with bi-allelic SNPs using plink:

plink --vcf VCFFILENAME --double-id --snps-only \ --set-all-var-ids @:# --maf 0.000001 --make-bed \ --out PLINKFILENAME

Next, we combine the sequencing data from the 0.1X Mal'ta genome with the precomputed allele frequencies. To do so, we create a text file to specify that we will filter out all reads with a mapping quality <30, all nucleotides with a base quality <20 and we will trim 5 bases from each of the ends of every read.

echo -e 'MalTa_subsample.bam\tMalTa\tMalTa\t30\t20\t5'> bamlist.txt

We then run *addBams.py*. This procedure was completed in <1 min. Note that one could alternatively run *bam2plink.py* to obtain a plink file with pseudo-haploid calls from the BAM file.

frantk addBams listname=bamlist.txt \ freqpref=HGDP_hg19_genotypes_f \

newpref=HGDP_hg19_genotypes_f_WithBam nthr = 1

Using the merged data, we can explore the broad genetic affinities of the low-depth Mal'ta genome. We use the *autof3wfixed.R* wrapper (with 40 threads) to compute the 51 possible *f_3_*-statistics of the form *f_3_(Mal'ta, X; Yoruba)*, where *X* represents all the populations in the HGDP SNP array dataset. This run was completed in ∼20 s.

frantk autof3wfixed \ freqpref=HGDP_hg19_genotypes_f_WithBam h1 = MalTa \

target=Yoruba catfile=HGDP_hg19_genotypes_cat \

legfile=HGDP_hg19_genotypes_leg nthr = 40

*autof3wfixed.R* will output the plot shown in Figure 2A. Note that color and symbol information is stored in the *HGDP_hg19_genotypes_cat* and *HGDP_hg19_genotypes_leg* files, which we supply through the *catfile* and *legfile* options. These results replicate the finding in (Raghavan *et al.* 2014), that the population represented by the ∼24 ka Mal'ta individual was genetically most closely related to present-day Indigenous Americans, followed by present-day West Eurasians.

Raghavan *et al.* (2014) showed that the ancestors of Indigenous Americans most likely descended from an admixture event between an East Asian-related population and a Mal'ta-related population. We can test whether the Mal'ta individual is a better proxy for the ancient North Eurasian population that contributed to the ancestry of Indigenous Americans than present-day West Eurasians are. We run the *autoDwfixed.R* wrapper (with 40 threads) to compute all possible *D*-statistics of the form *D(Karitiana, Han; X, Yoruba)*, where *X* represents all the populations in the HGDP dataset, including the Mal'ta individual. This run was completed in ∼20 s.

frantk autoDwfixed \ freqpref=HGDP_hg19_genotypes_f_WithBam \ h1 = Karitiana \

h2 = Han h4 = Yoruba \ catfile=HGDP_hg19_genotypes_cat \

legfile=HGDP_hg19_genotypes_leg nthr = 40

*autoDwfixed.R* will output the plot shown in Figure 2B. These results support that the ancient population that mixed with an East Asian-related population to give rise to the ancestors of Indigenous Americans was more closely related to the ancient Mal'ta individual than to present-day populations around the world.

In Figure 2B, we observed that, although the French are not the best proxy for the non-East Asian-related ancestry in Indigenous Americans, *D(Karitiana, Han; French, Yoruba)* deviated significantly from *D = 0* (*Z*∼14.9). This pattern is most likely due to shared ancestry between the Mal'ta individual and present-day French (Lazaridis *et al.* 2014). We can explore how Mal'ta-related admixture in Indigenous Americans affects this statistic by computing admixture-corrected *f_4_*-statistics (Reich *et al.* 2012). We use the *autof4subtr.R* wrapper to compute

f4(Han,Karitiana; French,Yoruba)-cf4(Han,Mal'ta; French,Yoruba)1-c,

assuming an admixture proportion *c* range between 0% and 50%, with 2.5% increases. This run was completed in ∼10 s.

frantk autof4subtr \ freqpref=HGDP_hg19_genotypes_f_WithBam \ h1 = Han \

h2 = Karitiana h3 = French h4 = Yoruba x=MalTa \ minp = 0 maxp=.5 pstep = 0.025 \ catfile=HGDP_hg19_genotypes_cat \

legfile=HGDP_hg19_genotypes_leg nthr = 40

Figure 2C shows that subtracting *c *≥* *0.375 Mal'ta-related admixture results in *f_4_*-statistics with *|Z|*<3. These results are consistent with the previous estimate of 14–38% Mal'ta-related admixture in Indigenous Americans (Raghavan *et al.* 2014).

We can use the *Freqs2Treemix.py* script to convert the allele frequency file to the treemix input format (Pickrell and Pritchard 2012). First, we create a file with a list of populations of interest (one per line). Note that all populations should be present in the *_pop* file. Then we run *Freqs2Treemix.py*, which will output two sets of treemix files, one with all sites and one with transversions polymorphisms only (useful for aDNA). This procedure was completed in ∼30 s.

echo "San

MbutiPygmy

Yoruba

Mandenka

Papuan

Melanesian

Han

Dai

French

Italian

Sardinian

Orcadian

MalTa

Karitiana

Surui" > poi

frantk Freqs2Treemix \ freqpref=HGDP_hg19_genotypes_f_WithBam \

tmpref=hgdp_malta_tm popsofint=poi

Finally, we run treemix following the parameters in Raghavan *et al.* (2014).

#Compute the number of SNPs that should be included in each autosomal 5 Mb-block.

a=`zcat hgdp_malta_tm_ALL_tm.gz | wc -l`

nsnps=`echo "5000000/(2881033286/"$a")” | bc`



#Run treemix with 0 and 1 migrations

treemix -i hgdp_malta_tm_ALL_tm.gz -o tm_ALL_res_0mig -k "$nsnps" -noss \

-global -root San -m 0 -seed 012345

treemix -i hgdp_malta_tm_ALL_tm.gz -o tm_ALL_res_1mig -k "$nsnps" -noss \

-global -root San -m 1 -seed 112345

Treemix admixture graphs in Figure 2, D and E suggest that the Mal'ta individual forms a clade with West Eurasians, but contributes to the ancestry of present-day Indigenous Americans.

## Conclusion

FrAnTK is a toolkit that streamlines a set of common analyses that rely on allele frequency-based statistics, and makes them accessible to users that are less familiar with computer programming. We reduce memory and computing times by precomputing allele frequencies, thus allowing researchers to explore their own datasets with reduced computational resource requirements. Notably, the automated wrappers and plotting functionality in FrAnTK allow the user to carry out complex exploratory analyses and produce publication-ready visual representations with single-line commands and minimal data manipulation. Thus, we consider an appropriate protocol would comprise an initial exploration using the tools in FrAnTK, followed by the application of model-based strategies such as those implemented in qpWave and qpGraph (Reich *et al.* 2012).

## Data availability

FrAnTK and its documentation are freely available in github.com/morenomayar/FrAnTK.

Supplementary material is available at *G3* online.

## Supplementary Material

jkab357_Supplementary_Data
