# Supplementary material for: FrAnTK: a Frequency-based Analysis ToolKit for efficient exploration of allele sharing patterns in present-day and ancient genomic datasets
Source: G3 (Bethesda). 2021 Oct 13;12(1):jkab357. doi: 10.1093/g3journal/jkab357 (PMC8727993; doi:10.1093/g3journal/jkab357)
Supplement: jkab357_Supplementary_Data [file jkab357_supplementary_data.pdf]

Supplementary Information
FrAnTK: A Frequency-based Analysis ToolKit for efficient exploration of allele sharing patterns in present-day and ancient genomic datasets

1 Input and output file format description

In what follows, we describe the structure of the input and output files required and produced by FrAnTK.
Visit github.com/morenomayar/FrAnTK for a tutorial on how to run the different tools.

1.1 Input plink and _clust files

We start with a `bed-bim-fam` plink file (Chang *et al.*, 2015) containing genome-wide bi-allelic SNP data from multiple individuals from different populations. Alleles in the `.bim` file should be coded using ACGT and not numbers. This can be accomplished by running plink with the `--alleleACGT --make-bed` options. Individuals in the plink file are mapped to population names using a `_clust` file. This is a **tab-separated** table with four columns:

- Columns 1 and 2 correspond to the first two columns in the `.fam` file.
- Column 3 maps each individual to a population.
- Column 4 may be `0` or `1`. Set to `0` when an individual has diploid genotypes, and set to `1` when an individual has pseudo-haploid genotypes (standard for low-depth ancient DNA data). Note that the ploidy of all individuals in a population should be the same. If different values are specified, the ploidy of the population is set to the most frequent value (diploid for ties).

1.2 Precomputed allele frequency files

`BuildFreqs.py` creates four output files, all starting with a user-specified prefix:

1. `prefix_freqs.gz` contains the allele frequencies per population per site (one site per row). This is a tab-separated file with `5+3n+2` columns, where `n` is the number of populations in the `_clust` file. Columns 1-5 correspond to the physical coordinate information in the plink `.bim` file. The following columns are organised in per-population triplets. The first element of the triplet is the allele frequency of the allele in column 4. The second and third elements are the population allele counts of the alleles in columns 4 and 5, respectively. Each row has two additional elements that keep track of the number of copies of each of the alleles (columns 4 and 5), in the complete dataset.
2. `prefix_pop` contains the population names in the dataset. Triplets in `prefix_freqs.gz` are ordered according to this file.
3. `prefix_regions` is an extended `BED` file (not to be confused with a plink `.bed` file) containing the coordinates of each site in the dataset (0-based start, 1-based end intervals).
4. `prefix_chrs` is a tab-separated file with five columns that keeps track of the contig sizes:
   - Column 1 contains the ordered names of the chromosomes in the dataset
   - Columns 2 and 3 contain the coordinates of the first and last SNPs for each chromosome.
   - Columns 4 and 5 contain the line number of the first and last SNP in each contig (useful for subsetting and sorting).

1.3 Single statistics output

By default, the output of the `get*.py` scripts is written into a directory named `./res`. One can specify the output directory using the `resdir` option. All output files are tab-separated and the following columns depending on the script that is run:

- The first columns contain the names of the populations in each test:

  - h1, h2 for pairwise distances
  - h1, h2, target for f_3 (Patterson *et al.*, 2012)
  - h1, h2, h3, h4 for D and f_4 (Patterson *et al.*, 2012)
  - h1 (A), h2 (B), h3 (C), h4 (O), x (the B+C=X admixed population) for f_4-ratio (Patterson *et al.*, 2012)
  - h1, h2, h3, h4, x (the population that admixes into h2) for admixture/contamination-corrected f_4 (Reich *et al.*, 2012)

These are followed by:

- the statistic of interest (e.g., f_3-statistics)
- the standard error as estimated from a weighted block-jackknife (Patterson *et al.*, 2012). The user can specify the length of the blocks (in bp). Note that for each contig, the first block starts on the first SNP and not in bp 1.
- Z-score (statistic of interest/std.error)
- the number of snps used for computation
- the number of blocks used for the block-jackknife
- a logical flag (used for plotting, see below)
- a category (also used for plotting, see below)
- D and f_4 output files contain two additional columns with the expected counts of ABBA and BABA sites (Patterson *et al.*, 2012).

1.4 Automated wrapper output

Automated `auto*.R` wrappers will output individual results into a new directory. These scripts also produce a concatenated results text file and a pdf plot for each run, *e.g.*. Figure 2a,b. Every run will produce a plot with default plotting parameters, but this plot can be easily modified by providing `category` and `legend` files using the `catfile` and `legfile` options, respectively.

1. A `category` file is a tab-separated file with two columns specifying a 'category' for each population:
   - Column 1 corresponds to the populations that are included in the panel (see `_clust` file definition above)
   - Column 2 is a user-specified category. The default category value 'Other' is assigned to populations that are not present in this file.
2. A `legend` file is a tab-separated file with four columns that map categories in the `catergory` file to plotting colors and symbols:
   - Column 1 is a category name (specified in the `category` file)
   - Column 2 is an R color name (outer color for filled symbols)
   - Column 3 is an R color name (inner color for filled symbols).
   - Column 4 is an R symbol number.

   Random colors and symbols are chosen for categories that are not present in the `legend` file.

The user has the option to define which populations are to be included in the plot by editing the concatenated output .txt file. This can be accomplished by setting the 'Plot' column to FALSE (capitalisation is important) to exclude a given test. After having run the automated wrappers once, the concatenated results file can be reused to explore different plotting options using the `resfile` argument.

1.5 Adding sequencing data

We provide two options for combining sequencing data in a BAM/CRAM file (Li *et al.*, 2009) with a reference dataset. For each BAM/CRAM file, we sample one random allele at every SNP position included in the reference dataset to generate pseudo-haploid calls (calls giving rise to tri-allelic sites are set to missing). `addBams.py` takes as input a tab-separated file with the six columns that map BAM/CRAM files to population names and filtering parameters:

- Column 1 is the path to a BAM/CRAM file.
- Column 2 is the individual ID of the BAM/CRAM file.
- Column 3 is the population ID of the BAM/CRAM file. This field can be used to pool data from multiple individuals into a population.
- Column 4 defines a minimum read mapping quality filter.
- Column 5 defines a minimum base quality filter.
- Column 6 is the number of bases to trim from both ends of the reads. This filter is particularly useful when including aDNA data with *postmortem* damage (Briggs *et al.*, 2007).

`addBams.py` will create new output files, similar to those produced by `BuildFreqs.py`. Alternatively, one can use the `bam2plink.py` script which will also generate pseudo-haploid calls from a BAM/CRAM file, but these will be stored into separate plink files. This is useful when the user is preparing their data for other analyses that do not rely on allele frequencies. We note that these tools assume that the order of the contigs in the BAM/CRAM files (in the bam header) corresponds to the order of the contigs in the precomputed allele frequency files (`_freqs.gz`, `_chrs`, `_regions`). We provide a separate script `CheckContigOrder.R` that can be used to synchronise both files.

Supplementary Table 1: **Summary of the main tools included in this FrAnTK+ release**. Tools are categorised according to their main functionality. This information can be obtained by running the frantk master wrapper. Visit github.com/morenomayar/FrAnTK for tool-specific detailed usage instructions.

Tool name	Functionality	Brief description
`frantk`	Master wrapper	Provides a straightforward, centralised way to call and get help for all main tools
`BuildFreqs.py`	Precomputing allele frequencies from a SNP variation dataset	Compute allele frequencies from a plink file for frequency-based-statistics.
`getf3.py`	Compute allele frequency-based statistics	Compute f_3-statistics of the form $f_3(h1, h2; target)$ following (Patterson *et al.*, 2012).
`getD.py`	Compute allele frequency-based statistics	Compute D statistics of the form $D(h1, h2; h3, h4)$ following (Patterson *et al.*, 2012).
`getF4.py`	Compute allele frequency-based statistics	Compute f_4 statistics of the form $f_4(h1, h2; h3, h4)$ following (Patterson *et al.*, 2012).
`getF4Ratio.py`	Compute allele frequency-based statistics	Compute f_4-ratios of the form $f4(h1, h4; x, h3)/f_4(h1, h4; h2, h3)$ following (Patterson *et al.*, 2012).
`getF4subtr.py`	Compute allele frequency-based statistics	Compute admixture subtracted f_4 statistics of the form $(f_4(h1, h2; h3, h4) - p_{adm} * f_4(h1, x; h3, h4))/(1 - p_{adm})$ following (Reich *et al.*, 2012).
`getPWdist.py`	Compute allele frequency-based statistics	Compute pairwise distance between 2 populations, defined as the average of $p_{h1} * q_{h2} + q_{h1} * p_{h2}$ over all sites.

`getEnhD.py`	Compute allele frequency-based statistics	Compute enhanced D-statistics of the form $D(h1, h2; h3, h4)$ following (Meyer *et al.*, 2012). We restrict to sites where $h4$ is invariant for one allele. Works better with many sites and not too many individuals/populations with very different ancestries in $h4$.
`getDstrat.py`	Compute allele frequency-based statistics	Compute D-statistics of the form $D(h1, h2; h3, h4)$, on sites where there are $(minder, maxder]$ derived alleles in $(h1+h2+h3+h4)$. Similar to (Prufer *et al.*, 2012), but we pick segregating sites in $h1 + h2 + h3 + h4$.
`getDstrat2.py`	Compute allele frequency-based statistics	Compute D-statistics of the form $D(h1, h2; h3, h4)$, on sites where there are $(minder, maxder]$ derived alleles in the complete panel (freqpref). Similar to (Prufer *et al.*, 2012), but we pick segregating sites in the complete panel.
`getDtrip.py`	Compute allele frequency-based statistics	Compute the three possible arrangements of $D(h1, h2; h3, h4)$.
`autof3wfixed.R`	Automated computation and visualisation of multiple related statistics	Compute f_3 statistics of the form $f_3(h1, h2; target)$. Fix two out of $(h1, h2, target)$ and loop over the remaining one.
`autoPWf3wfixed.R`	Automated computation and visualisation of multiple related statistics	Compute f_3 statistics of the form $f_3(h1, h2; target)$, for two samples x and y and get pairwise comparisons. x and y will be fixed as $h1$, while $h2$ will loop over all other populations in the panel, $target$ is fixed as well.
`autoDwfixed.R`	Automated computation and visualisation of multiple related statistics	Compute D-statistics of the form $D(h1, h2; h3, h4)$. Fix three out of $(h1, h2, h3, h4)$ and loop over the remaining one. Run with f4=1 to compute f_4 instead of D.
`autof4subtr.R`	Automated computation and visualisation of multiple related statistics	Compute admixture subtracted f_4 statistics of the form $(f_4(h1, h2; h3, h4) - p_{adm} * f_4(h1, x; h3, h4))/(1 - p_{adm})$ following (Reich *et al.*, 2012). We compute f_{4subtr} for values of p_{adm} within the range $[p_{min}, p_{max}]$, with step size p_{step}.
`autoPWdistwfixed.R`	Automated computation and visualisation of multiple related statistics	Compute pairwise distance between 2 populations, defined as the average of $p_{h1} * q_{h2} + q_{h1} * p_{h2}$ over all sites. Fix one out of $(h1, h2)$ and loop over the remaining one.
`autoDEnhwfixed.R`	Automated computation and visualisation of multiple related statistics	Compute enhanced D-statistics of the form $D(h1, h2; h3, h4)$ following (Meyer *et al.*, 2012). We restrict to sites where $h4$ is invariant for one allele. Works better with many sites and not too many individuals/populations with very different ancestries in $h4$. Fix $h4$ and two out of $(h1, h2, h3)$ and loop over the remaining one.

`autoDstrat.R`	Automated computation and visualisation of multiple related statistics	Compute D-statistics of the form $D(h1, h2; h3, h4)$ on sites with a given number of derived alleles. Similar to (Prufer *et al.*, 2012). We compute D for sites with $[minder, maxder]$ (step size=$dstep$) derived alleles. Set strattype=1 or strattype=2 to count derived alleles in $h1, h2, h3, h4$ (1) or in the complete panel (2).
`addBams.py`	Merging sequencing data with a SNP variation dataset	Add sequencing data in one or more bam files to a precomputed frequency file (output from BuildFreqs.py). For each bam file, one random allele is sampled at each site present in the frequency file. (see complementary bam2plink.py and BuildDummyFreqs.py scripts)

References

Briggs,A.W., *et al.* (2007) Patterns of damage in genomic DNA sequences from a Neandertal. *Proc Natl Acad Sci U S A.*, **104**, 14616-14621.

Chang,C., *et al.* (2015) Second-generation PLINK: rising to the challenge of larger and richer datasets. *Gigascience*, **4**, 10.1186/s13742-015-0047-8.

Li,H., *et al.* (2009) The Sequence Alignment/Map format and SAMtools. *Bioinformatics*, **25**, 2078-2079.

Meyer,M., *et al.* (2012) A High-Coverage Genome Sequence from an Archaic Denisovan Individual. *Science*, **338**, 222-226.

Patterson,N., *et al.* (2012) Ancient admixture in human history. *Genetics*, **192**, 1065-1093.

Prüfer,K., *et al.* (2013) The complete genome sequence of a Neanderthal from the Altai Mountains. *Nature*, **505**, 43-49.

Reich,D., *et al.* (2012) Reconstructing Native American population history. *Nature*, **488**, 370-374.
